# Effect of Flavoring with Rosemary, Lemon and Orange on the Quality, Composition and Biological Properties of Olive Oil: Comparative Study of Extraction Processes

**DOI:** 10.3390/foods12061301

**Published:** 2023-03-18

**Authors:** Hassiba Chahdoura, Zeineb Mzoughi, Borhane E. C. Ziani, Yasmine Chakroun, Mohamed Ali Boujbiha, Safia El Bok, Manel Ben M’hadheb, Hatem Majdoub, Wissem Mnif, Guido Flamini, Habib Mosbah

**Affiliations:** 1 Unité de Recherche UR17ES30 “Génomique, Biotechnologie et Stratégies Antivirales”, Institut Supérieur de Biotechnologie de Monastir, Université de Monastir, BP74, Avenue Tahar Hadded, Monastir 5000, Tunisia; hassiba_chahdoura@yahoo.fr (H.C.); benmhadhebmanel@yahoo.fr (M.B.M.); 2 Laboratory of Interfaces and Advanced Materials, Faculty of Sciences of Monastir, University of Monastir, Monastir 5000, Tunisia; mzoughizeineb.lima@gmail.com (Z.M.); hatemmajdoub.fsm@gmail.com (H.M.); 3 Centre de Recherche Scientifique et Technique en Analyses Physico-Chimiques CRAPC, Tipaza 42000, Algeria; ziani.ensa@gmail.com; 4 Laboratory of Bioresources: Integrative Biology and Valorization (BIOLIVAL), Higher Institute of Biotechnology of Monastir, University of Monastir, Avenue TaherHadded BP 74, Monastir 5000, Tunisia; yasminechakroun9@gmail.com (Y.C.); boujbihamohamedali@gmail.com (M.A.B.); mosbah_habib@yahoo.fr (H.M.); 5 Laboratory of Biodiversity, Biotechnologies and Climate Change (LR11/ES09), Department of Biology, Faculty of Sciences of Tunis, University of Tunis El-Manar, Tunis 2092, Tunisia; safia1elbok@yahoo.fr; 6 Department of Chemistry, Faculty of Sciences at Bisha, University of Bisha, P.O. Box 199, Bisha 61922, Saudi Arabia; 7 Diparitmento di Farmacia, Via Bonanno 6, 56126 Pisa, Italy; guido.flamini@unipi.it; 8 Interdepartmental Research Centre “Nutraceuticals and Food for Health”, University of Pisa, Via del Borghetto 80, 56124 Pisa, Italy

**Keywords:** flavored olive oil, chemical compositions, aroma volatiles, antioxidant properties, pharmacological properties

## Abstract

The goal of this work was to investigate the impact of the flavoring of some aromatic plants/spices, including rosemary (R), lemon (L) and orange (O) at the concentration of 5% and 35% (*w*/*w*) added by 2 methods (conventional maceration and direct flavoring), on quality attributes, chemical changes and oxidative stability of extra virgin olive oil (EVOO). Six flavored oils were obtained (EVOO + O, O + O, EVOO + R, O + R, EVOO + L and O + L). The physicochemical parameters (water content, refractive index, acidity and peroxide value, extinction coefficient, fatty acids, volatile aroma profiles, Rancimat test, phenols and pigments composition) of the flavored oils were investigated. Based on the results obtained, it was observed that flavoring with a conventional process provided increased oxidative stability to the flavored oils, especially with rosemary (19.38 ± 0.26 h), compared to that of unflavored oil. The volatile profiles of the different flavored oils revealed the presence of 34 compounds with the dominance of Limonene. The fatty acid composition showed an abundance of mono-unsaturated fatty acids followed by poly-unsaturated ones. Moreover, a high antioxidant activity, a significant peripheral analgesic effect (77.7% of writhing inhibition) and an interesting gastroprotective action (96.59% of ulcer inhibition) have been observed for the rosemary-flavored oil. Indeed, the flavored olive oils of this study could be used as new functional foods, leading to new customers and further markets.

## 1. Introduction

According to the European Food Safety Authority (EFSA), olive oil is widely produced and consumed as one of the main functional foods [1]. It is also part of the Mediterranean diet due to its delicious taste and aroma, as well as its nutritional properties [2]. The latter are mainly related to its fatty acids composition, in particular to the high content of oleic acid, and also to the balanced ratio between saturated and polyunsaturated fatty acids [3]. In addition, olive oil contains significant amounts of natural antioxidants and is considered essential in the prevention of many diseases [4]. Tunisia is one of the largest olive oil producing countries. It is the largest African exporter and ranks fourth in the world after Spain, Italy and Greece [5,6]. At the moment, its consumption is becoming increasingly popular among consumers, mainly in Northern Europe, the United States and Canada [7]. However, most of these potential consumers are not familiar with the different applications of olive oil and may be more willing to purchase olive oil preparations flavored with other ingredients related to the Mediterranean diet. In parallel, a great effort has recently been made to improve the quality of olive oils produced in Tunisia. Obtaining good quality flavored oils, as well as increasing the use of olive oil among non-traditional consumers, would further enhance this precious agricultural product. In addition, it has been shown that the incorporation of bioactive ingredients leads to an increase in the phenols rate in olive oil with an increase in its oxidative stability and its antioxidant activity. Thereafter, the consumption of such flavored olive oils can help to avoid several chronic diseases [8]. Among the ingredients that could be used for the production of flavored olive oil, there are certainly aromatic herbs and spices [9]. In fact it is well-known that these ingredients help maintain the nutritional value of the oil and increase its shelf life [10]. Aromatic plants and spices are fundamental ingredients in Mediterranean cuisine (i.e., rosemary in grilled meat or chicken, lemon in salads or soups and orange in desserts/coffee). According to some previous investigations, lemon [11], chili peppers [12,13,14], mint and thyme [15], orange [16], mugwort [17], lavender and sage [10], basil [18], rosemary [19,20] and saffron [21] have been used for the preparation of flavored olive oil to improve its sensory qualities and satisfy the consumers. In addition, numerous biological activities are generally attributed to these enriched oils or some of their components, including antioxidant and antimicrobial properties [22,23]. In general, different extraction methods have been developed, such as the conventional [24] and ultrasound-assisted extraction ones [18] for the preparation of flavored olive oils, with the aim of preserving the antioxidant molecules from degradation and improving the quality of the resulting enriched oils. Maceration is the oldest and most widely used method of flavoring oil, and it involves simply mixing herbs, spices or fruits into the oil. It must be followed by filtration to remove turbidity and solids to obtain a clear, flavored olive oil [25]. Other approaches have also been used, such as the direct addition of essential oils [26] or herbs to the crushed olives before the malaxation step [27]. Therefore, the aims of this study were to produce a new range of olive oils flavored with selected Tunisian aromas (rosemary, lemon and orange). Furthermore, different flavoring methods have been compared to verify their effect on the physicochemical modifications of the final products with respect to the starting one. In addition, the antioxidant and some pharmacological activities were also evaluated.

## 2. Materials and Methods

### 2.1. Plant Material

The unflavored olive oils were obtained from “Chemlali” olive fruits (*Oleaeuropaea* L.) and picked manually in olive groves located in the region of Bennan (Ksibet El Mediouni, Monastir- Tunisia). The fresh fruits of oranges and lemons (*Citrus sinensis* and *C.limon,* Rutaceae) originated from the Cap-Bon region (Peninsula in far north-eastern Tunisia). Rosemary seeds (*Rosmarinusofficinalis* L., Lamiaceae) were purchased in a local market (Monastir, Tunisia). The aromatic plants/spice were identified by a taxonomist.

### 2.2. Flavored Olive Oils Preparation

Two enrichment methods were adopted:

#### 2.2.1. Aromatization by Conventional Maceration Process

The flavored olive oil preparation was performed as mentioned in a previous paper [10] with slight modifications. Fruits/spices were prepared as a freeze-dried powder and then added to an olive oil at the concentration of 5% (*w*/*w*). The mixture was stirred by mechanical stirring (300 rpm) at room temperature (20 ± 2 °C) for 4 h. The blends were stored in tightly closed stainless steel bottles for 2 weeks in an aerated and dark place to avoid any oxidation phenomena. After the maceration step, the samples of olive oils flavored by conventional maceration (FOOCM) were recovered by sieving and centrifugation (4500 rpm, 20 min), followed by filtration. The process was performed in triplicate.

#### 2.2.2. Aromatization by Direct Addition of Aromatic Fruits or Spice

The enrichment method is based on the direct addition of fresh aromatic fruits (orange/lemon) or spices (rosemary) to the olives (0.35 kg/kg). The plant materials were crushed using a mixing grinder SM1 Type, Retsch GmbH (Schneider Industries, Magdeburg, Germany) for 30 min before the malaxation step during the extraction process of the olive oil (under mechanical stirring, 300 rpm, at room temperature for 4 h). In the final phase, the mixture was centrifuged (4500 rpm, 20 min) and then filtrated to obtain flavored olive oils by direct aromatization (FOODA), after removing the wastewater. This process was also performed in triplicate.

In parallel, samples of unflavored olive oil and the control oils were treated in the same way. The unflavored and flavored olive oils were stored in the dark until analysis.

The flow chart of the experimental plan is shown in Figure S1 (Electronic Supplementary Material).

### 2.3. Physical and Chemical Characteristics of Flavored Oils

The water content and free acidity index were determined according to the ISO 729-1985 standard, specific for oils from seeds and oleaginous fruits. The peroxide value was determined following the AFNOR NF T60-220 standard, and extinction coefficients (K_232_ and K_270_) were determined according to AOCS (1998). The refractive index was used to measure the sample purity [28], and the density was measured by a pycnometer at 25 °C.

### 2.4. Phytochemical Composition of Flavored Oils

#### 2.4.1. Chlorophyll and Carotenoids Content

Pigments quantifications was performed following the technique described by [29]. The contents of chlorophylls and carotenoids were expressed as mg of pheophytin “a” and lutein per kg of oil, respectively.

#### 2.4.2. Fatty Acids Profile

The fatty acids of the oil were determined on the basis of the procedure described by [30]. Analysis of the fatty acid composition was achieved with a gas chromatograph system. The fatty acid methyl ester (FAME) composition was determined by converting the oil to fatty acid methyl esters by the addition of 1 mL of hexane to 40 mg of oil followed by 200 μL of sodium methoxide (2 M). At that time, the mix was heated in a bath (50 °C) for a few seconds followed by adding 200 μL of HCl (2N). The top layer (1 μL) was injected into a Model 5890 Series II GC (Hewlett Packard Headquarters in Palo Alto, California, United States) equipped with a flame ionization detector (FID) and a polar capillary column (HP–Innowax polyethylene glycol 0.25 mm internal diameter 30 m length and 0.25 m thick film). The detector temperature was set to 275 °C, and the column temperature was programmed starting from 150 °C maintained for 1 min and increased at a rate of 15 °C/ min up to 200 °C and then with a rate of 2 °C/ min up to 250 °C and detained for 4 min. The whole time was 45 min. Peaks of FAME were identified by comparing their retention time with individual FAME standards. Data were expressed as a percentage of individual fatty acids in the lipid fraction.

#### 2.4.3. Fourier Transform Infrared (FT-IR) Spectra Analysis

Attenuated total reflectance Fourier transform infrared (ATR-FTIR) spectra of olive oil samples were obtained with a Fourier Transform Infrared spectrophotometer (FTIR system spectrometer, PerkinElmer, MA, USA) in the absorbance mode from 4000 to 400 cm^−1^ (mid infrared region). Spectral data were analyzed with the Origin Pro 8 software program [31].

#### 2.4.4. Volatile Compounds Analyses

The volatile compounds were analyzed by solid phase micro-extraction coupled with gas chromatography/mass spectrometry (SPME-GC/MS). Identification was carried out by the comparison of their retention times, linear retention indices (LRI), mass spectra matching against commercial libraries (NIST 2014 and ADAMS) and, when available, with those of pure standards processed under the same conditions [32].

#### 2.4.5. Test of Rancimat

This experiment permits the investigation of the oxidation stability as described by [33]. The oils were thermo-oxidized using Rancimat 743 equipment (Metrohm, Filderstadt, Germany) at 120 °C.

#### 2.4.6. Phenolic Compounds Extraction

##### Preparation of the Methanolic Extracts of Flavored Oils

Briefly, 10 g of olive oil was extracted using 10 mL of a methanol–water mixture (80:20, *v*/*v*) as extraction solvent. The extracts were obtained by stirring for 1min on an ultra turrax homogenizer. After centrifugation (10 min at 5000 rpm), the methanol phase was recovered and stored at 4 °C for 24 h until use.

##### Total Phenolics and *o*-Diphenols Contents

Total phenols content was estimated using the [34] method. Gallic acid was employed as standard for the calibration curve, and results were expressed as mg of gallic acid equivalents (GAE) per kg of extract. The *o*-diphenols rate was measured according to [35] and expressed as mg caffeic acid equivalents (CAE) per kg of extract.

### 2.5. Biological Evaluation

#### 2.5.1. Antioxidant Activities

Each aqueous-methanolic extract of the oil samples was screened for DPPH and ABTS^+^ radical-scavenging abilities [36], reducing power, as well as ß-carotene bleaching inhibition assays [37]; the EC50 value (mg/mL extract) of each assay was calculated by interpolation.

#### 2.5.2. Pharmacological Investigations

##### Animals

Albino Wistar of both sexes weighting 180–200 ± 20 g and albino Swiss male mice 25–30 g, provided by the Pasteur Institute of Tunis, Tunisia, were used to evaluate their gastroprotective and analgesic activities. Animals were handled in strict compliance with the widely accepted ethical guidelines of the Tunisian Society for the Care and Use of Laboratory Animals, and the study was approved by the University of Monastir Ethical Committee (Approval No: CER-SVS 007/2020 ISBM). The animals were housed under standard laboratory conditions with a 12 h light–dark cycle at constant temperature (22 ± 2 °C) and humidity (55 ± 5%) and were provided with food and water ad libitum before the experiments.

##### Analgesic Activity

The analgesic activity was evaluated by the abdominal constriction test with acetic acid (writhing test) according to the method of [38]. The number of writhings was counted over a period of 30 min. The percentages of writhing inhibition were obtained with the following formula:

% Inhibition = [(number of writhes (control) − number of writhes (test)) / (number of writhes (control))] × 100 (1)

##### Ethanol-Induced Gastric Damage

The gastroprotective activity of the different flavored oils has been studied in gastric ulcer induced by HCl/EtOH [39]. The surface was examined for the presence of lesions and to measure their extent. The summed length of the lesions along the stomach was recorded (mm) as lesion index.

### 2.6. Statistical Analysis

All experiments were performed in triplicate. All values were expressed as means ± standard deviation (SD). Data were elaborated using the software Statistical Package for the Social Sciences (SPSS), version 20.0. Quantitative differences were interpreted by ANOVA; *p*-values of 0.05 or less were considered statistically significant.

Principal Components Analysis (PCA) was applied as an unsupervised classification method of pattern recognition. The number of dimensions to retain for data analysis was assessed by their respective eigenvalues, by the Cronbach’s alpha parameter and also by the total percentage of variance explained by the number of selected components. The number of plotted dimensions was chosen to allow for meaningful interpretations.

The PCA was used to evaluate a possible correlation between the different biological activities and the physicochemical parameters of the various flavored olive oils.

## 3. Results and Discussion

### 3.1. Impact of the Aromatization Treatment on the Quality Parameters of Olive Oil

Two different methods of flavoring olive oil were tested using rosemary, lemon and orange. The traditional method consists of extracting the natural aroma by infusing it into the oil. The second method, generally less used, is based on the extraction of the natural aroma by adding the plant material to the olive paste just before the malaxation step. The latter method gave worse results than the former. Indeed, lower free acidity and peroxide values were obtained using the traditional method, as presented in Table 1. Furthermore, the flavoring with the conventional process has provided flavored oils with higher oxidative stability and excellent quality (Table 2).

In addition, it has made it possible to obtain products with a higher content of polyphenols and pigments. On the contrary, the lemon-flavored oil (O + L) produced with the second method contained two volatile aroma compounds, nonanal and (E)-2-dodecene, not identified in the corresponding flavored oils obtained with the traditional process. Moreover, in addition to an increase in antioxidant activities, the traditional method determined a significant antinociceptive potential for peripheral analgesic activity and a good gastroprotective effect of the different flavored oils (data presented below).

### 3.2. Physicochemical Characteristics of Flavored Oils

The physicochemical changes observed for unflavored and flavored oils are summarized in Table 1. Remarkable, statistically significant differences were found depending on the added flavoring aroma (*p* < 0.05).

The orange flavoring resulted in an increase in water content of 0.04%, 0.94% and 1.21% for the OC, EVOO + O and O + O oils, respectively. Most likely, this important content could be explained by the addition of a quantity of water coming from the aroma during the oil extraction procedure [40]. However, the flavoring caused the water content to decrease when rosemary was used as a flavoring agent, passing from 0.15 to 0.09%, which can improve the stability of the resulting product.

The refractive index values of all samples ranged between 1.4679 and 1.4702. These data complied with the values cited by the IOC (2015) and the Codex Stan 33-2011 standard, which varies from 1.4677 to 1.4705 for extra virgin olive oil.

In addition, the density values of the different samples ranged between 0.9126 and 0.9144 (Table 1) and complied with the IOC and the CODEX STAN 33-2011 standard (between 0.910 and 0.916 for EVOO). This outcome was in agreement with the previously mentioned density values (0.9106, 0.9201 and 0.9173, respectively, for virgin olive oil, lemon flavored olive oil and rosemary flavored olive oil) [10]. Based on these data, it can be hypothesized that all flavored oils can be considered “pure” due to better filtration.

Free acidity is generally the first parameter that allows the evaluation of the quality of an olive oil; it is a factor that provides information on its degradation and, therefore, on its quality [41]. As summarized in Table 1, all samples showed remarkably low acidity values, ranging from 0.08 to 0.21%. The unflavored olive oil (OC) has the highest acidity content (0.21%), which is in any case less than 0.80%, the limit established by the IOC (2011) for extra virgin olive oil, confirming the high quality of the oil, which was obtained from healthy olives and in ideal and highly controlled conditions. Lemon flavored olive oil (EVOO + L) had the lowest acidity content (0.08%). The addition of spices, such as rosemary, caused a decrease in the acidity index. This decrease can be considered a quality performance indicator for the industrial marketing. These results are in good agreement with previous studies [42,43].

The peroxide value is another very important parameter that monitors the oxidative processes in the early stages. As presented in Table 1, the peroxide index was higher in orange flavored olive oil (EVOO + O), indicating more formation of primary oxidation products. Additionally, the peroxide values are higher than the limit set by the IOC (2015) for extra virgin olive oil (20 meq of O_2_/kg). These values are between 20 meq O_2_/kg for lemon flavored oil (O + L) and 86.66 meq O_2_/kg for orange flavored oil (EVOO + O). The increase of this index is reflected in a high oxidation of olive oils and was probably caused by various factors [44].

The oxidation state of an olive oil can be estimated by evaluating the specific extinction coefficients at 232 and 270 nm. Oxidation leads to the formation of conjugated dienes, which absorb at 232 nm. Pronounced oxidation can result in by-products that absorb at 270 nm. As reported in Table 1, the specific absorbance values (K232, K270) are within the limits of the IOC (2015) for an EVOO (K232 ≤ 2.5; K270 ≤ 0.20). In detail, the lemon flavored olive oil (EVOO + L) showed the lowest extinction coefficient at 232 nm (2.25 ± 0.07) while the highest coefficient was obtained for the olive oil flavored with rosemary (EVOO + R). On the other hand, at 270 nm, all values ranged from 0.09 to 0.20. These results are comparable to those obtained by Dabbou et al. [45], who indicated that K232 coefficient varied from 1.60 to 2.80 and K270 coefficient ranged between 0.10 and 0.20. Therefore, it can be noted that flavoring with herbs and/or spices can affect the quality indices of virgin olive oils [27].

Chlorophylls and carotenoids play a major role in oxidative stability due to their natural action as antioxidants in the dark and pro-oxidants in the light; they are also mainly responsible for the color of olive oil, which ranges from yellow-green to greenish-gold. Moreover, these pigments are also important for the stability of olive oil [16]. The carotenoid and chlorophyll content of the different flavored olive oils is presented in Table 1.

Carotenoids reached high levels, between 8.61 mg/Kg for the control oil (OC) and 31.77 mg/Kg for the orange flavored one (EVOO + O). In the case of the two control oils (RLC and OC) and for those flavored with lemon and rosemary (EVOO + L, O + L, EVOO + R and O + R), discrepancies were found with respect to previously published data [10]. The chlorophyll contents varied between 21.06 mg/Kg for the control oil (OC) and 47.96 mg/Kg for the rosemary flavored olive oil (EVOO + R), values higher than those obtained by [10]. Both contents were also higher than those previously reported using other aromatic plants [1]. These pigments are also considered to be indicators of olive oil freshness, nutritional value, authenticity and antioxidant activities. According to Criado et al., the addition of hot water during oil extraction (malaxation step) favors a degradation of chlorophylls by the action of chlorophyllase [46]. However, these differences in pigment concentrations may be due to several parameters (i.e., environmental conditions, degree of fruit ripeness, harvest time and agricultural practices adopted) [47]; other factors that may be responsible for the differences are of technological nature, such as the method applied to extract the oil or the storage and packaging conditions of the finished product [13].

### 3.3. Oxidative Stability Evaluation (Rancimat Method)

This test permits to evaluate the stability of olive oils to oxidation and to determine the Rancimat induction time (expressed in hours), which corresponds to the time during which the fat has resisted oxidative stress. As summarized in Table 2, adding rosemary to olive oil significantly improved its oxidative stability. This stability can be linked to the richness of rosemary in natural antioxidants, such as polyphenols and *o*-diphenols or metabolites with a high capacity to prevent lipid oxidation. This strict correlation between phenolic content and oxidative stability was previously observed [48]. The oxidative stability of olive oil depends not only on the characteristics of olives, such as the variety and quality, but also on agricultural practices (i.e., cultivation area and harvesting time that contribute to determining its content of antioxidants, such as tocopherols, phenols and carotenes. The extraction process and storage conditions can also have a potential effect on its shelf life [49]. Orange flavored olive oil had an intermediate induction time (11 h) while the lowest value was noted for lemon flavored olive oil (8 h). This decrease in oxidative stability could be due to the water activity of these samples [50].

### 3.4. Fatty Acids Profile

The fatty acids content of flavored olive oils was characterized by GC–MS analysis. The results are presented in Table 3 and indicate the presence of 16 compounds, the main one being oleic acid (C18:1), followed by an appreciable quantity of linoleic acid (C18:2n6c). Oleic acid has a fundamental role in the prevention of cardiovascular diseases and is known to be very important in in the development of neurons [51]. Among the saturated fatty acids, the dominant component was palmitic acid (15.90–18.59%). In summary, a predominance of monounsaturated fatty acids (MUFA, 66.68–68.42%), a low percentage of polyunsaturated fatty acids (PUFA, 8.70–11.01%) and a moderate percentage of saturated fatty acids (SFA, 18.59–22.21%) can be noted. Here in, it can be concluded that all the flavored oils could also have a beneficial effect on health, given their richness in MUFA, known for their effect on the LDL and HDL-cholesterol levels.

### 3.5. Volatile Component Characteristics

A total of 34 volatile components were characterized (Table 4), representing more than 99.5% of the total emission. The volatile profile of control oils (RLC + OC) consisted mainly of non-terpene derivatives. The main constituents emitted by the two oils was *(E)*-2-hexenal (60.7 and 53.0%, respectively). This aldehyde that characterizes both control samples originates from the enzymatic degradation of lipids (free fatty acids such as linoleic and linolenic acids) through the lipoxygenase pathway [52]. Moreover, the aromatization method had a significant effect on the volatile compositions. The main variations observed concerned the richness of EVVO + R in limonene (60.5%) while the O + R was rich in 1,8-cineole (59.2%). Other important constituents of the latter flavored oil were camphor and α-pinene, which reached similar percentages.

Additionally, the effect of aromatization on the composition was also observed for the lemon flavored olive oils (EVOO + L and O + L); the presence of two new volatile compounds (nonanal and *(E)*-2-dodecene) was evidenced for the O + L sample. Thus, the major component of the lemon flavored olive oils was limonene (62.1% for EVOO + L and 60.2% for O + L). Conversely, it was noted that the two different flavoring processes have no substantial effect on the volatile components of the orange flavored oils (EVOO + O and O + O). Indeed, they showed a very similar emission pattern, with limonene as the major volatile (92.7 and 91.4%, respectively).

Esters, ketones and aldehydes were the main oxygenated aroma compounds. The presence of rather high levels of certain esters, aldehydes and terpenoids can give the final products a strong and pleasant smell of fruits, citrus and flowers [53].

### 3.6. FTIR Analysis

The FTIR spectra of rosemary flavored olive oil and control oil (RLC) are shown in Figure S2A. They were quite similar, both having as main bands those due to the absorption peaks of common triglycerides, the main component of edible fats and oils.

Indeed, the two samples showed a broad and intense stretching peak at about 3005 cm^−1^, characteristic for the stretching vibration of =C-H. Moreover, strong absorption bands were observed in the region of 3000–2800 cm^−1^ caused by the C–H stretching. Additionally, the stretching vibrations of the methylene (–CH2–) and methyl (–CH3) groups can be observed at 2921 and 2852 cm^−1^, respectively. The presences of methylene and methyl groups are also confirmed by their bending vibrations at 1461 cm^−1^ and 1375 cm^−1^.

Furthermore, the signal around 1743 cm^−1^ is due to the C=O double bond stretching of carboxylic acids or ketones. In addition, deformation and bending of C–H and stretching of C–O resulted in peaks in the 1500–650 cm^−1^ region [54]. The differences between the two spectra were clearly small; these data suggest that IR spectroscopy does not allow discriminating between flavored and unflavored olive oils.

### 3.7. Phenolic Compounds

Phenolic compounds play an important role in the characterization and nutritional value of olive oils. These compounds are mainly responsible for the stability of olive oils during storage and heating [55]. The contents of polyphenols and *o*-diphenols in control and flavored oils are presented in Table 5. The polyphenol content of control olive oils was 649.35 and 648.86 mg GAE/kg for the RLC and OC, respectively. These values conformed to the standard recommended by IOC (2015) for EVOO (153–694 mg/kg). Several studies have indicated that phenols, which can be present in free, bound or esterified forms, are the main antioxidant substances present in olive oils [56]. Thus, results indicated a significant increase in the polyphenol content of the flavored EVOO (*p* < 0.05). Rosemary flavored olive oil (EVOO + R) revealed the highest content of polyphenols (1185.98 mg GAE/kg) while the lowest value was found for the lemon flavored one (O + L), with a content of 383.76 mg GAE/kg. One of the possible causes of this increase could be the hydrolysis of some phenolic substances, such as oleuropein, which are broken down into hydroxytyrosol [57]. In addition, this increase could be explained by the enrichment due to the addition of rosemary, a spice having a rather high polyphenols content, as previously reported [10]. Contrary to the above, the addition of lemon caused a significant decrease (*p* < 0.05) in the polyphenol content. This phenomenon could be due to the degradation of polyphenols as a result of their antioxidant activity and/or to the activity of polyphenol oxidases responsible for the oxidation of polyphenols [58]. These results contrast with those published by Ayadi et al. (2009) for lemon and rosemary flavored olive oils (130 mg GAE/kg and 170 mg GAE/kg, respectively). These differences could be explained by the ripening stage of the olives, by the method of oil extraction or by the aromatization techniques adopted [59].

The contents of *o*-diphenols, expressed as mg of CAE/kg, varied between 111.16 and 170.85 g CAE/kg (Table 5). As in a previous study [13], where the contents of polyphenols and *o*-diphenols were interconnected, this trend is also confirmed in the present study. A particularly high concentration of *o*-diphenols was observed for the rosemary-flavored olive oil (EVOO + R; 170.85 mg CAE/kg). On the contrary, the olive oil flavored with lemon (O + L) exhibited the lowest content, with 111.16 g CAE/kg. In all the flavored oils, the phenols content is increased, thus improving their nutraceutical properties.

### 3.8. Biological and Pharmacological Assessment

#### 3.8.1. Antioxidant Properties

Olive oil has been shown to have powerful antioxidant, antibacterial and antifungal activities [60]. Polyphenols are well-known for their antioxidant abilities as radical scavengers and for their possible beneficial roles in human health, such as reducing the risk of cancer, cardiovascular disease and other pathologies [61].

For these reasons, the antioxidant potential of the flavored olive oils was evaluated using four complementary techniques, including their ability to scavenge DPPH and ABTS free radicals, their power to reduce iron (III) and their capacity to prevent inhibition of β-carotene bleaching. As reported in Table 5, significant differences (*p* ˂ 0.05) were observed for the different flavored oil samples.

Indeed, rosemary-flavored olive oil (EVOO + R) exhibited outstanding antioxidant activities on DPPH (EC50 = 0.14 mg/mL), ABTS^+^. (EC50 = 5.30 mg/mL), reducing power (EC50 = 0.04 mg/mL) and β-carotene bleaching (EC50 = 0.69 mg/mL). This strong anti-free radical potential could be correlated with the high concentration of total polyphenols [62]. In addition, these findings could be attributed to the fact that rosemary is rich in powerful nonpolar antioxidants, which allow it to inhibit the oxidation of linoleic acid coupled to β-carotene [63,64]. Previous studies report that this strong activity may be due to the presence of the hydroxyl groups of phenolic compounds that can act as electron donors [65]. This observation is confirmed by the behavior of the lemon-flavored olive oil (O + L), poor in phenols, which had the lowest EC50 values in all antioxidant assays.

#### 3.8.2. Antinociceptive Activity

The acetic acid-induced writhing reaction has been widely used as a screening tool for assessing peripheral analgesic activity due to its sensitivity and simplicity [66]. After the administration of flavored olive oils, a dose-dependent and significant inhibition of the amount of abdominal writhing was observed. As presented in Table 6, at doses of 7.5 g/Kg, the (EVOO + R) and (EVOO + O) samples significantly reduced the amount of writhing by 77.70% and 63.06%, respectively. This significant decrease could be due to the high content of various fatty acids, such as linoleic, palmitic and stearic acids, known for their analgesic action [67]. Indeed, even the two control virgin olive oils (RLC; OC) at the same dose of 7.5 g/Kg, significantly reduced, albeit to a lesser extent, the amount of writhing (52.70% and 51.89%, respectively). These results are in good agreement with those reported by other researchers on the antinociceptive activity of extra virgin olive oil [68]. Acetylsalicylate of lysine (ASL), used as a reference peripheral acting drug, at the dose of 200 mg/kg reduced the amount of writhing by 63.83%. These results showed that EVOO + R exhibited an important antinociceptive activity which, in addition to its fatty acids, could also be related to its important content of phenolic compounds [69]. According to Ito et al. [70], acetic acid indirectly induces the release of endogenous mediators (prostaglandins PGE2 and PGF2α, substance P and cytokines IL-1,TNF-α and IL-8 sensitive to non-steroidal anti-inflammatory drugs. Animals pretreated with flavored olive oils modified their acetic acid-induced nociceptive response in a dose-dependent manner, suggesting that the analgesic action of polyphenols could inhibit the release of these mediators.

#### 3.8.3. Gastroprotective Effect

Table 6 and Figure S2B summarize the anti-ulcerogenic properties of the flavored olive oils, investigated with the HCl/EtOH induced gastric damage model. This method has been widely used to measure the preventive properties of several compounds on mucosal damage. Gastric damage induced by ethanol is associated with its ability to dissolve the gastric mucus layer as well as to stimulate the secretion of histamine, pepsin and H^+^ ions, and it is characterized by marked and diffused areas of hemorrhage in the stomach [39]. As shown in Figure S2B, HCl/EtOH produced a gastric mucosal injury with severe bleeding and a lesion index of 58.16 mm in the untreated group. Pretreatment with flavored olive oils at 2.5 and 7.5 mg/Kg produced a significant decrease in the intensity of damage to the gastric mucosa compared to the control group. Notably, the treatment of rats with the EVOO + R and O + R samples at doses of 7.5 mg/Kg produced the highest decrease in gastric hemorrhage and the lesion index was inhibited by 96.59 and 90.40%, respectively (Table 6). These results are comparable to those of omeprazole (30 mg/Kg) and (60 mg/Kg). Indeed, the two classic anti-ulcer drugs ranitidine (histamine H2 receptor antagonist) and omeprazole (proton pump inhibitor) showed a significant gastroprotective activity with a percentage of inhibition of gastric lesions of 65.76 and 91.83%, respectively.

In the case of the EVOO + O and O + O samples, the activity was lower, but the protection was still significant, showing 81.89 and 78.31% inhibition, respectively. These differences could be explained by the higher amount of antioxidants in the rosemary [71]. As a result, EVOO + R may have a good potential to be used as a gastroprotective agent.

### 3.9. Principal Components Analysis

In order to investigate the correlation between the chemical profiles and the biological activities (antioxidant, analgesic and gastroprotective) of the different flavored olive oils, simultaneously considering the data obtained for all parameters, a principal component analysis (PCA) was performed. Only two dimensions have been plotted because including additional dimensions would not allow for a meaningful interpretation. As shown in Figure S2C, the horizontal axis of the PCA explained 21% of the total variance while the vertical axis further 29%. Overall, the graph showed that rosemary flavored olive oil is strongly correlated with the aromatic compounds content (i.e., limonene, camphor, α-pinene, etc.), as well as with the other phytochemicals (chlorophyll, polyphenols, etc.) and fatty acids (C16:0, C18:0, C20:0, etc.). Likewise, these levels are positively correlated with antioxidant activities (DPPH, ABTS, FRAP and β-carotene), pharmacological activities (analgesic and gastro-protective) and the oxidative stability (Rancimat test).

## 4. Conclusions

The present investigation provides information on the preparation of flavored EVOOs, new possible functional foods. The study focused on the impact of flavoring with rosemary, lemon and orange on the physico-chemical characterizations and biological activities of six flavored olive oils. The effect of different flavoring methods (conventional process and/or direct addition of aromatic plant material) was also evaluated. Aromatization by conventional process conferred the higher oxidative stability and excellent flavored oils purity. For olive oil flavored with rosemary, on the other hand, low values of free acidity and peroxides were found. The fatty acid composition revealed richness in monounsaturated fatty acids, followed by polyunsaturated ones. Oleic acid (C18:1) was the main acid in all flavored oils, followed by linoleic acid (C18:2n6c) and palmitic acid (C16:0). The Rancimat test indicated that the addition of rosemary to olive oil significantly improved its oxidative stability (induction time = 19 h), which could be correlated to the richness of rosemary in natural antioxidants (polyphenols and *o*-diphenols). Furthermore, the methanol extract prepared from rosemary-flavored oil (EVOO + R) has shown considerable potential for use in phytotherapy. Indeed, it exhibited excellent antioxidant capabilities. Moreover, a significant peripheral antinociceptive action of EVOO + R, (77.70% of writhing inhibition), associated to a notable gastroprotective effect, was observed.

Further works are needed to confirm these data and assess the long-term oxidative stability of the flavored oils.

## Figures and Tables

**Table 1:** Physicochemical characteristics of the control and different flavored EVOO.

Properties	Control		EVOO + O	O + O	EVOO + R	O + R	EVOO + L	O + L
	RLC	OC						
Water content (%)	0.15 ± 0.04 ^a^	0.04 ± 0.00 ^ab^	0.94 ± 0.05 ^c^	1.21 ± 0.03^c^	0.09 ± 0.02^a^	0.13 ± 0.09^ab^	0.82 ± 0.15 ^abc^	0.90 ± 0.08 ^bc^
Refractive index	1.4679 ± 0.002 ^a^	1.4700 ± 0.001 ^a^	1.4701 ± 0.001 ^a^	1.4691 ± 0.002 ^a^	1.4699 ± 0.001 ^a^	1.4702 ± 0.001 ^a^	1.4680 ± 0.002 ^a^	1.4689 ± 0.004 ^a^
Density (g)	0.9144 ± 0.001 ^a^	0.914 ± 0.001 ^a^	0.9136 ± 0.002 ^a^	0.9127 ± 0.004 ^a^	0.9133 ± 0.002 ^a^	0.9133 ± 0.001 ^a^	0.9139 ± 0.001 ^a^	0.9126 ± 0.002 ^a^
Acidity (% oleic acid)	0.19 ± 0.006 ^c^	0.21 ± 0.002 ^c^	0.11 ± 0.005 ^ab^	0.20 ± 0.005 ^c^	0.11 ± 0.005 ^ab^	0.16 ± 0.003 ^bc^	0.08 ± 0.01 ^a^	0.10 ± 0.004 ^a^
Peroxide index (meq of O_2_ per kg)	23.33 ± 0.004 ^a^	22.97 ± 0.01 ^ab^	86.66 ± 0.002 ^f^	22.66 ± 0.002 ^cd^	23.33 ± 0.005 ^cd^	30.66 ± 0.002 ^e^	24.66 ± 0.002 ^d^	20.00 ± 0.01 ^bc^
Extinction coefficients								
K_232_	2.40 ± 0.07 ^a^	2.42 ± 0.05 ^b^	2.39 ± 0.07 ^b^	2.45 ± 0.05 ^b^	2.45 ± 0.04 ^b^	2.42 ± 0.03 ^b^	2.39 ± 0.01 ^b^	2.25 ± 0.07 ^b^
K_270_	0.15 ± 0.01 ^c^	0.17 ± 0.05 ^e^	0.16 ± 0.04 ^d^	0.18 ± 0.05 ^f^	0.09 ± 0.05 ^a^	0.10 ± 0.04 ^b^	0.20 ± 0.09 ^h^	0.19 ± 0.04 ^g^
Pigments composition (mg/kg)								
Total carotenoids	9.20 ± 0.01 ^a^	8.61 ± 0.002 ^a^	31.77 ± 0.06 ^e^	20.94 ± 0.02 ^g^	22.88 ± 0.01 ^f^	17.63 ± 0.02 ^d^	14.83 ± 0.04 ^c^	12.53 ± 0.02 ^b^
Total chlorophylls	22.23 ± 0.03 ^a^	21.06 ± 0.03 ^b^	21.73 ± 0.04 ^a^	21.10 ± 0.02 ^a^	47.96 ± 0.05 ^e^	33.15 ± 0.03 ^d^	34.30 ± 0.02 ^d^	31.57 ± 0.03 ^c^

Results are presented as the average ± SD (*n* = 3). Different letters on the same line indicate significant differences (*p* < 0.05) with LSD test. EVOO: extra virgin olive oil; RLC: rosemary and lemon control; OC: orange control; EVOO + O: extra virgin olive oil with orange; O + O: olive with orange; EVOO + R: extra virgin olive oil with rosemary; O + R: olive with rosemary; EVOO + L: extra virgin olive oil with lemon; O + L: olive with lemon.

**Table 2:** Induction time of EVOO, control and different flavored EVOO (Rancimat method).

Type of EVOO	Control		EVOO + O	O + O	EVOO + R	O + R	EVOO + L	O + L
	RLC	OC						
Induction time (hours)	13.10 ± 0.45 ^e^	13.20 ± 0.51 ^de^	11.77 ± 0.60 ^cd^	10.39 ± 0.35 ^bc^	19.38 ± 0.26 ^f^	18.83 ± 0.36 ^f^	9.22 ± 0.18 ^b^	7.29 ± 0.26 ^a^

Results are presented as the average ± SD (*n* = 3). Different letters on the same line indicate significant differences (*p* < 0.05) with LSD test. EVOO: extra virgin olive oil; RLC: rosemary and lemon control; OC: orange control; EVOO + O: extra virgin olive oil with orange; O + O: olive with orange; EVOO + R: extra virgin olive oil with rosemary; O + R: olive with rosemary; EVOO + L: extra virgin olive oil with lemon; O + L: olive with lemon.

**Table 3:** Fatty acids profile (relative percentage, %) of the control and different flavored EVOO.

Fatty Acids	Control		EVOO + O	O + O	EVOO + R	O + R	EVOO + L	O + L
	RLC	OC						
Pentadecanoic acid(C15:0)	0.24 ± 0.01 ^a^	0.26 ± 0.01 ^a^	-	-	-	-	-	-
Palmitic acid(C16:0)	16.80 ± 0.08 ^a^	15.9 ± 0.10 ^b^	17.96 ± 0.06 ^d^	16.92 ± 0.14 ^b^	18.59 ± 1.62 ^e^	17.42 ± 1.12 ^c^	17.72 ± 1.05 ^d^	17.30 ± 2.08 ^c^
Palmitoleic acid(C16:1)	2.30 ± 0.01 ^b^	2.6 ± 0.05 ^d^	2.22 ± 0.01 ^c^	2.07 ± 0.03 ^a^	2.09 ± 0.16 ^b^	2.07 ± 1.02 ^a^	2.07 ± 0.89 ^a^	2.4 ± 0.36 ^c^
Margaric acid(C17:0)	-	-	0.03 ± 0.01^a^	-	-	-	0.03 ± 0.01^a^	-
Oleic acid (C18:1)	66.12 ± 1.11 ^b^	64.86 ± 2.18 ^a^	65.58 ± 1.19 ^b^	65.29 ± 1.25 ^b^	65.50 ± 2.04 ^b^	65.28 ± 2.45 ^b^	65.45 ± 2.15 ^b^	64.31 ± 1.69 ^a^
Linoleic acid(C18:2n6c)	9.98 ± 0.06 ^b^	10.06 ± 0.04 ^c^	10.79 ± 0.08 ^d^	10.02 ± 0.56 ^c^	8.37 ± 1.01 ^a^	10.15 ± 0.25 ^c^	8.89 ± 1.25 ^a^	10.45 ± 0.95 ^d^
Stearic acid (C18:0)	2.15 ± 0.01 ^b^	2.05 ± 0.02 ^a^	2.51 ± 0.01 ^b^	2.03 ± 0.39 ^a^	2.91 ± 0.28 ^b^	2.56 ± 0.48 ^b^	2.90 ± 1.08 ^b^	2.56 ± 0.65 ^b^
Arachidic acid(C20:0)	0.19 ± 0.01 ^a^	0.23 ± 0.01 ^a^	0.42 ± 0.01 ^c^	0.43 ± 0.02 ^c^	0.47 ± 0.12 ^e^	0.23 ± 1.02 ^b^	0.45 ± 0.05 ^d^	0.43 ± 0.15 ^c^
Gondoic acid (C20:1 n-9)	-	-	0.18 ± 0.01^a^	-	0.23 ± 0.08	-	0.20 ± 0.09 ^b^	-
Behenic acid(C22:0)	0.12 ± 0.01 ^a^	0.11 ± 0.01 ^a^	0.11 ± 0.01 ^a^	0.12 ± 0.03 ^a^	0.13 ± 0.05 ^a^	0.12 ± 0.02 ^a^	0.12 ± 0.02 ^a^	0.12 ± 0.02 ^a^
Tricosanoic acid(C23:0)	-	-	0.02 ± 0.01 ^a^	-	0.03 ± 0.06 ^a^	-	-	-
Lignoceric acid(C24:0)	0.06 ± 0.01 ^b^	0.04 ± 0.01 ^a^	0.07 ± 0.01 ^c^	0.06 ± 0.01	0.07 ± 0.02 ^b^	0.06 ± 0.01	0.09 ± 0.01 ^b^	0.06 ± 0.01 ^a^
Hexacosanoic acid (C26:0)	-	-	-	-	0.01 ± 0.01^a^	-	0.09 ± 0.02^b^	-
Saturated fatty acid (SFA) Monounsaturated fatty acid (MUFA) Polyunsaturated fatty acid (PUFA)	19.56 ± 0.08 ^b^ 68.42 ± 1.09 ^b^ 9.98 ± 0.02 ^b^	18.59 ± 0.13 ^a^ 67.46 ± 0.23 ^a^ 10.06 ± 0.02 ^b^	21.10 ± 1.11 ^d^ 66.68 ± 2.05 ^a^ 11.01 ± 1.01 ^b^	19.56 ± 1.14 ^b^ 67.65 ± 2.17 ^a^ 10.02 ± 0.65 ^b^	22.21 ± 0.13 ^e^ 67.82 ± 2.18 ^a^ 8.70 ± 1.01 ^a^	20.39 ± 1.08 ^c^ 67.35 ± 2.36 ^a^ 10.15 ± 1.28 ^a^	21.40 ± 0.19 ^d^ 67.72 ± 0.85 ^a^ 9.14 ± 1.08 ^a^	20.35 ± 1.52 ^c^ 66.71 ± 2.15 ^a^ 10.45 ± 1.02 ^b^

Results are presented as the average ± SD (*n* = 3). Different letters on the same line indicate significant differences (*p* < 0.05) with LSD test. EVOO: extra virgin olive oil; RLC: rosemary and lemon control; OC: orange control; EVOO + O: extra virgin olive oil with orange; O + O: olive with orange; EVOO + R: extra virgin olive oil with rosemary; O + R: olive with rosemary; EVOO + L: extra virgin olive oil with lemon; O + L: olive with lemon, - not determined.

**Table 4:** Volatiles (%) of the control and different flavored EVOO.

N°	Constituents	LRI	Control		EVOO + O	O + O	EVOO + R	O + R	EVOO + L	O + L
			RLC	OC						
1	*(E)*-2-hexenal	856	60.7 ± 0.6 ^d^	53 ± 0.5 ^e^	0.1 ± 0.01 ^a^	0.1 ± 0.01 ^a^	1.5 ± 0.10 ^c^	1.00 ± 0.1 ^b^	-	-
2	α-thujene	933	-	-	-	-	-	-	0.3 ± 0.1 ^a^	0.3 ± 0.1 ^a^
3	α-pinene	941	-	-	1.3 ± 0.1 ^a^	1.5 ± 0.1 ^b^	9.5 ± 0.1 ^e^	10.6 ± 0.1 ^f^	4.2 ± 0.1 ^c^	4.4 ± 0.1 ^d^
4	Camphene	955	-	-	-	-	6.9 ± 0.1 ^a^	7.5 ± 0.1 ^b^	-	-
5	Sabinene	977	-	-	1.5 ± 0.1 ^a^	1.9 ± 0.1 ^a^	-	-	-	-
6	β-pinene	982	-	-	-	-	5.1 ± 0.1 ^a^	5.6 ± 0.1 ^a^	14.8 ± 0.2 ^b^	15.6 ± 0.2 ^b^
7	Myrcene	993	-	-	3.4 ± 0.1 ^c^	3.9 ± 0.1 ^c^	1.6 ± 0.1 ^a^	2.1 ± 0.1 ^b^	5 ± 0.1 ^d^	5.3 ± 0.1 ^d^
8	3,7-decadiene *	999	4.5 ± 0.1 ^a^	12.6 ± 0.2 ^b^	-	-	-	-	-	-
9	*(Z)*-3-hexenyl acetate	1008	3.4 ± 0.1 ^a^	9.8 ± 0.1 ^b^	-	-	-	-	-	-
10	1-hexyl acetate	1010	1.3 ± 0.08	-	-	-	-	-	-	-
11	δ-3-carene	1013	-	-	0.2 ± 0.05 ^a^	0.2 ± 0.06 ^a^	-	-	-	-
12	α-terpinene	1020	-	-	-	-	-	-	0.1 ± 0.00	-
13	p-cymene	1028	-	-	-	-	0.2 ± 0.03 ^a^	0.4 ± 0.04 ^b^	0.2 ± 0.03 ^a^	0.2 ± 0.02 ^a^
14	Limonene	1032	22.4 ± 0.6 ^b^	8.2 ± 0.4 ^a^	92.7 ± 0.9 ^e^	91.4 ± 0.8 ^e^	60.5 ± 0.6 ^c^	-	62.1 ± 0.5 ^d^	60.2 ± 0.6 ^c^
15	1,8-cineole	1034	-	-	-	-	-	59.2 ± 0.9	-	-
16	*(E)*-β-ocimene	1052	0.5 ± 0.08 ^b^	-	0.1 ± 0.01 ^a^	-	-	-	-	-
17	γ-terpinene	1063	1.2 ± 0.3 ^b^	-	-	-	0.2 ± 0.04 ^a^	0.3 ± 0.04 ^a^	11.9 ± 0.8 ^c^	12.8 ± 0.9 ^c^
18	Terpinolene	1090	-	-	0.1 ± 0.01 ^a^	0.1 ± 0.02 ^a^	-	-	0.4 ± 0.02 ^b^	0.3 ± 0.01 ^a^
19	Linalool	1101	-	-	0.4 ± 0.03 ^a^	0.6 ± 0.04 ^b^	0.3 ± 0.02 ^a^	-	-	-
20	Nonanal	1104	1.8 ± 0.03 ^b^	-	-	0.1 ± 0.01 ^a^	-	0.3 ± 0.02 ^a^	-	5.6 ± 0.6 ^b^
21	Camphor	1144	-	-	-	-	10.8 ± 0.2 ^a^	10.6 ± 0.9 ^a^	-	-
22	Borneol	1168	-	-	-	-	1.6 ± 0.02 ^b^	0.9 ± 0.01 ^a^	-	-
23	4-terpineol	1179	-	-	-	-	0.1 ± 0.01	-	-	-
24	α-terpineol	1191	-	-	-	-	-	0.5 ± 0.03	-	-
25	Decanal	1204	-	-	0.1 ± 0.01 ^a^	0.1 ± 0.01 ^a^	-	0.2 ± 0.02 ^a^	-	-
26	*(E)*-2-dodecene	1205	1.8 ± 0.08 ^b^	-	-	-	0.1 ± 0.01 ^a^	-	-	8.4 ± 0.25 ^c^
27	1-ocytyl acetate	1213	2 ± 0.16	-	-	-	-	-	-	-
28	Neral	1240	-	-	-	-	-	-	0.3 ± 0.06 ^a^	0.3 ± 0.05 ^a^
29	Geranial	1271	-	-	-	-	-	-	0.4 ± 0.07 ^a^	0.4 ± 0.06 ^a^
30	Bornyl acetate	1286	-	-	-	-	-	0.3 ± 0.06	-	-
31	Neryl acetate	1366	-	-	-	-	-	-	-	0.1 ± 0.01
32	β-caryophyllene	1419	-	-	-	-	0.6 ± 0.05 ^b^	0.4 ± 0.03 ^a^	-	-
33	*(E)*-geranylacetone	1456	-	1.6 ± 0.26	-	-	-	-	-	-
34	*(E,E)*-α-farnesene	1508	0.2 ± 0.01 ^a^	0.7 ± 0.08 ^b^	0.1 ± 0.01 ^a^	-	-	-	-	-
	Monoterpene hydrocarbons		24.1 ± 0.9 ^b^	8.2 ± 0.74 ^a^	99.2 ± 0.9 ^e^	99 ± 0.85 ^e^	84 ± 0.87 ^d^	26.5 ± 0.6 ^c^	99.1 ± 0.9 ^e^	99 ± 0.92 ^e^
	Oxigenated monoterpenes		-	-	0.4 ± 0.05 ^a^	0.6 ± 0.06 ^b^	13.3 ± 0.2 ^d^	71.5 ± 0.8 ^e^	0.7 ± 0.75 ^a^	0.8 ± 0.82 ^bc^
	Sesquiterpene hydrocarbons		0.2 ± 0.02 ^b^	0.7 ± 0.06 ^e^	0.1 ± 0.01 ^a^	-	0.6 ± 0.05 ^d^	0.4 ± 0.03 ^c^	-	-
	Apocarotenoids		-	1.6 ± 0.02	-	-	-	-	-	-
	Non-terpene derivatives		75.5 ± 0.7 ^c^	89.4 ± 0.9 ^d^	0.2 ± 0.01 ^a^	0.3 ± 0.02 ^a^	1.6 ± 0.01 ^b^	1.5 ± 0.01 ^b^	-	-
	Non-terpene hydrocarbons		6.3 ± 0.06 ^b^	21 ± 0.19 ^c^	-	-	0.1 ± 0.01 ^a^	-	-	-
	Non-terpene aldehydes/ketones		62.5 ± 0.6 ^d^	58.6 ± 0.6 ^c^	0.2 ± 0.01 ^a^	0.3 ± 0.02 ^a^	1.5 ± 0.01 ^b^	1.5 ± 0.02 ^b^	-	-
	Non-terpene esters		6.7 ± 0.07 ^a^	9.8 ± 0.08 ^b^	-	-	-	-	-	-
	Total identified (%)		99.8 ± 0.9	99.9 ± 0.8	99.9 ± 0.9	99.9 ± 0.9	99.5 ± 0.8	99.9 ± 0.9	99.8 ± 0.8	99.9 ± 0.9

Results are presented as the average ± SD (*n* = 3). Different letters on the same line indicate significant differences (*p* < 0.05) with LSD test. LRI: linear retention indices on DB-5 column; *: unidentified isomer. EVOO: extra virgin olive oil; RLC: rosemary and lemon control; OC: orange control; EVOO + O: extra virgin olive oil with orange; O + O: olive with orange; EVOO + R: extra virgin olive oil with rosemary; O + R: olive with rosemary; EVOO + L: extra virgin olive oil with lemon; O + L: olive with lemon, - not determined.

**Table 5:** Phenolic composition and antioxidant (EC_50_ values) activities of the control and different flavored EVOO.

		Control		EVOO + O	O + O	EVOO + R	O + R	EVOO + L	O + L
		RLC	OC						
Phenols composition	Total Phenolics content (mgGAE/kg)	649.35 ± 0.68 ^c^	648.86 ± 0.52 ^c^	761.97 ± 0.63 ^e^	680.08 ± 0.68 ^d^	1185.98 ± 0.75 ^g^	856.34 ± 0.48 ^f^	452.46 ± 0.28 ^b^	383.76 ± 0.23 ^a^
	Total *o*-diphenols content (mg CAE/kg)	132.03 ± 0.12 ^c^	131.76 ± 0.15 ^c^	145.10 ± 0.11 ^e^	140.94 ± 0.12 ^d^	170.85 ± 0.13 ^g^	158.07 ± 0.14 ^f^	122.32 ± 0.17 ^b^	111.16 ± 0.12 ^a^
Antioxidant activity (EC_50_, mg/mL)	DPPH scavenging ability	2.39 ± 0.12 ^d^	2.53 ± 0.07 ^d^	1.30 ± 0.01 ^a^	1.90 ± 0.08 ^b^	0.14 ± 0.02 ^a^	0.71 ± 0.01 ^c^	4.41 ± 0.16 ^e^	7.77 ± 0.19 ^f^
	ABTS	9.40 ± 0.06 ^c^	9.71 ± 0.09 ^c^	6.05 ± 0.23 ^c^	7.29 ± 0.13 ^a^	5.30 ± 0.64 ^ab^	5.54 ± 0.04 ^b^	10.85 ± 0.10 ^d^	12.43 ± 0.40 ^e^
	Reducing power	1.55 ± 0.01 ^d^	1.60 ± 0.08 ^d^	0.60 ± 0.01 ^a^	1.06 ± 0.03 ^a^	0.04 ± 0.001 ^b^	0.08 ± 0.04 ^c^	3.04 ± 0.03 ^e^	3.50 ± 0.17 ^f^
	β-carotene bleaching inhibition	4.49 ± 0.66 ^c^	4.89 ± 0.52 ^c^	1.61 ± 0.23 ^a^	2.01 ± 0.08 ^a^	0.69 ± 0.30 ^b^	1.02 ± 0.02 ^b^	5.87 ± 0.003 ^d^	6.23 ± 0.04 ^d^

Results are presented as the average ± SD (n = 3). Different letters on the same line indicate significant differences (*p* < 0.05) with LSD test. GAE: Gallic acid equivalent; CAE: Caffeic acid equivalent; EC_50_: Extract concentration corresponding to 50% of antioxidant activity or 0.5 of absorbance in reducing power assay; EVOO: extra virgin olive oil; RLC: rosemary and lemon control; OC: orange control; EVOO + O: extra virgin olive oil with orange; O + O: olive with orange; EVOO + R: extra virgin olive oil with rosemary; O + R: olive with rosemary; EVOO + L: extra virgin olive oil with lemon; O + L: olive with lemon.

**Table 6:** Analgesic and gastroprotective activities of control and different flavored EVOO.

**Analgesic Activity ^A^**			
**Groups**	**Concentration (mg/Kg)**	**Number of Writhes**	**Inhibition of Writhing (%)**
Control (saline solution)	-	76.50 ± 0.39	-
RLC (Control)	250	52.66 ± 3.51 *	28.82
	500	47.16 ± 4.37 *	36.26
	1000	35.00 ± 5.55 **	52.70
OC (Control)	250	53.66 ± 3.79 *	27.48
	500	47.50 ± 4.44 *	35.81
	1000	35.60 ± 5.68 **	51.89
EVOO + O	250	47.16 ± 5.45 *	36.26
	500	33.66 ± 6.06 **	54.50
	1000	27.33 ± 4.73 **	63.06
O + O	250	49.50 ± 5.45 *	33.10
	500	37.50 ± 5.82 *	49.32
	1000	30.50 ± 4.99 **	58.78
EVOO + R	250	37.50 ± 5.53 *	49.32
	500	23.33 ± 3.95 **	68.47
	1000	16.50 ± 3.11 **	77.70
O + R	250	43.83 ± 5.72 *	40.76
	500	36.66 ± 5.67 **	50.45
	1000	30.16 ± 5.00 **	59.23
EVOO + L	250	56.33 ± 3.97 *	23.87
	500	32.65 ± 0.56 *	32.65
	1000	37.40 ± 5.18 *	49.46
O + L	250	58.50 ± 4.57 *	20.94
	500	52.83 ± 4.38 *	28.60
	1000	40.80 ± 5.07 *	44.86
Reference drug (ASL)	200	27.66 ± 2.83 **	63.83
**Gastroprotective Activity**			
**Groups**	**Concentration (mg/Kg)**	**Ulcer Index (mm)**	**Ulcer Inhibition (%)**
Control (Vehicle; 0.9% NaCl)	-	58.16 ± 2.14	-
RLC (Control)	500	40.00 ± 1.45 *	31.23
	1000	26.08 ± 1.74 **	55.15
OC (Control)	500	40.42 ± 1.12 *	30.51
	1000	26.33 ± 1.57 **	54.72
EVOO + O	500	37.5 ± 1.97 *	35.53
	1000	10.53 ± 0.66 **	81.89
O + O	500	38.83 ± 1.83 *	33.24
	1000	12.61 ± 1.94 **	78.31
EVOO + R	500	34.83 ± 0.75 *	40.11
	1000	1.98 ± 3.93 **	96.59
O + R	500	36.5 ± 1.87 *	37.25
	1000	5.58 ± 4.84 **	90.40
EVOO + L	500	41.42 ± 1.96 *	28.79
	1000	28.00 ± 1.67 **	51.86
O + L	500	42.67 ± 1.32 *	26.64
	1000	28.58 ± 2.35 **	50.86
Standard (Ranitidine)	50	19.91 ± 0.97 **	65.76
Standard (Omeprazole)	30	4.75 ± 0.76 **	91.83

Results are presented as the average ± SD (*n* = 6); *: *p* < 0.01 significant from control; **: *p* < 0.001 significant from control. ^A^ ASL: Acetylsalicylate of lysine. EVOO: extra virgin olive oil; RLC: rosemary and lemon control; OC: orange control; EVOO + O: extra virgin olive oil with orange; O + O: olive with orange; EVOO + R: extra virgin olive oil with rosemary; O + R: olive with rosemary; EVOO + L: extra virgin olive oil with lemon; O + L: olive with lemon.

## Data Availability

The data presented in this study are available on request from the first author.

## Supplementary Materials

The following supporting information can be downloaded at: https://www.mdpi.com/article/10.3390/foods12061301/s1, Figure S1: Flow chart of the experimental plan; Figure S2: (A): FTIR spectra of the (1) extra virgin olive oil (EVOO) and (2) extra virgin olive oil with rosemary (EVOO + R). (B): Effect of the different flavored EVOO (at a concentration of 7.5 mg/mL.) on gastric ulcer induced by EtOH/HClin Wistar rats. (C): Principal component analysis (PCA) based on the chemical composition and biological activities (antioxidant, enzymatic, analgesic and gastroprotective) of the different flavored olive oils. Object scores were highlighted for a better visualization.
