# Supplementary material for: Effect of Flavoring with Rosemary, Lemon and Orange on the Quality, Composition and Biological Properties of Olive Oil: Comparative Study of Extraction Processes
Source: Foods. 2023 Mar 18;12(6):1301. doi: 10.3390/foods12061301 (PMC10048770; doi:10.3390/foods12061301)
Supplement: Supplementary file 1 [file foods-12-01301-s001.zip › foods-2182446-SI.pdf]

Effect of Flavoring with Rosemary, Lemon and Orange on the Quality, Composition and Biological Properties of Olive Oil: Comparative Study of Extraction Processes

Hassiba Chahdoura [1], Zeineb Mzoughi [2], Borhane E. C. Ziani [3], Yasmine Chakroun [4], Mohamed Ali Boujbiha [4], Safia El Bok [5], Manel Ben M'hadheb [1], Hatem Majdoub [2], Wissem Mnif [6,*], Guido Flamini [7,8] and Habib Mosbah [4]

1 Unité de Recherche UR17ES30 "Génomique, Biotechnologie et Stratégies Antivirales", Institut Supérieur de Biotechnologie de Monastir, Université de Monastir, BP74, Avenue Tahar Hadded, Monastir 5000, Tunisia; hassiba_chahdoura@yahoo.fr (H.C.); benmhadhebmanel@yahoo.fr (M.B.M.)
2 Laboratory of Interfaces and Advanced Materials, Faculty of Sciences of Monastir, University of Monastir, Monastir 5000, Tunisia; mzoughizeineb.lima@gmail.com (Z.M.); hatemmajdoub.fsm@gmail.com (H.M.)
3 Centre de Recherche Scientifique et Technique en Analyses Physico-Chimiques CRAPC, Tipaza 42000, Algeria; ziani.ensa@gmail.com
4 Laboratory of Bioresources: Integrative Biology and Valorization (BIOLIVAL), Higher Institute of Biotechnology of Monastir, University of Monastir, Avenue TaherHadded BP 74, Monastir 5000, Tunisia; yasminechakroun9@gmail.com (Y.C.); boujbihamohamedali@gmail.com (M.A.B.); mosbah_habib@yahoo.fr (H.M.)
5 Laboratory of Biodiversity, Biotechnologies and Climate Change (LR11/ES09), Department of Biology, Faculty of Sciences of Tunis, University of Tunis El-Manar, Tunis 2092, Tunisia; safia1elbok@yahoo.fr or safia.elbok@fst.utm.tn
6 Department of Chemistry, Faculty of Sciences at Bisha, University of Bisha, P.O. Box 199, Bisha 61922, Saudi Arabia
7 Dipartimento di Farmacia, Via Bonanno 6, 56126 Pisa, Italy; guido.flamini@unipi.it
8 Interdepartmental Research Centre "Nutraceuticals and Food for Health", University of Pisa, Via del Borghetto 80, 56124 Pisa, Italy
* Correspondence: w_mnif@yahoo.fr

ADDITIONAL FIGURES (with Figure caption)

Figure S1. Flow chart of the experimental plan. EVOO: extra virgin olive oil; EVOO+O: extra virgin olive oil with orange; O+O: olive with orange; EVOO+R: extra virgin olive oil with rosemary; O+R: olive with rosemary; EVOO+L: extra virgin olive oil with lemon; O+L: olive with lemon.The tests were conducted in triplicate.

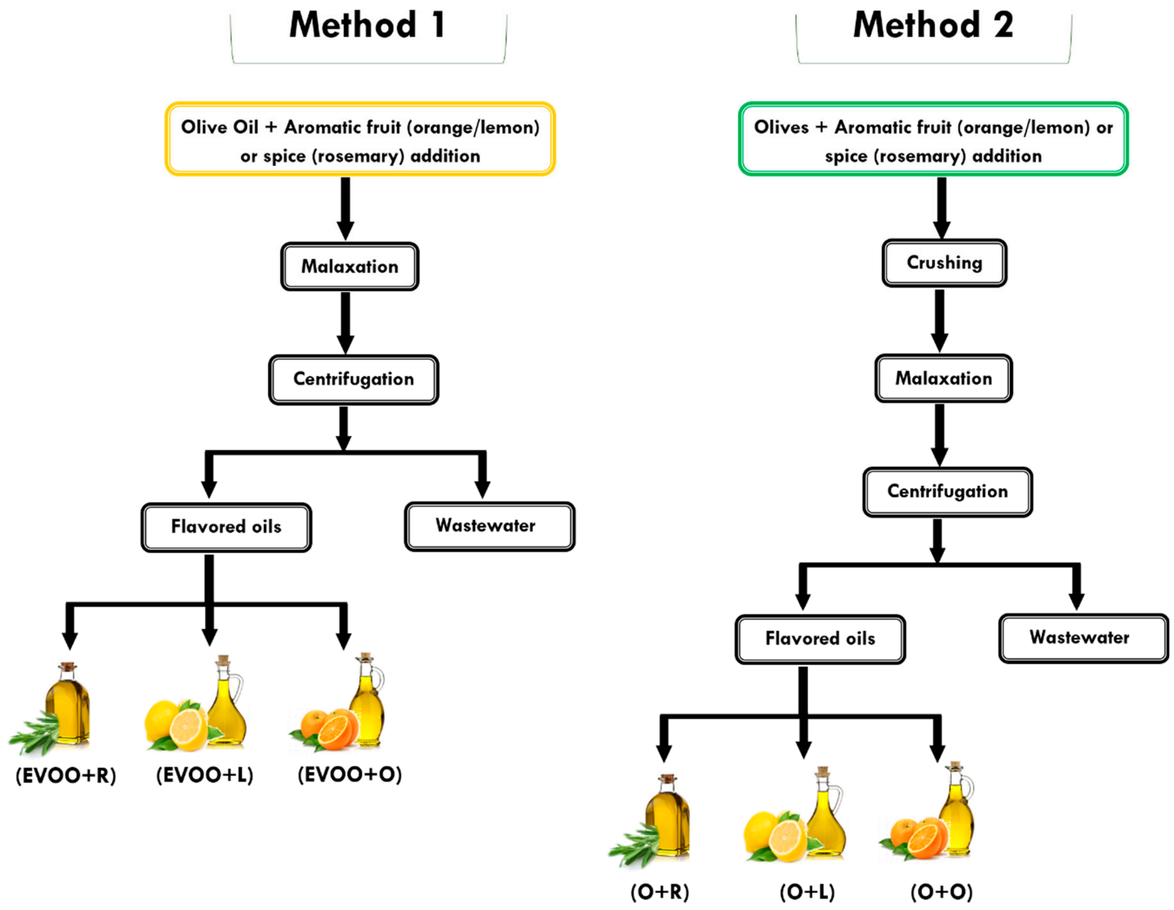

Figure S2. (A): FTIR spectra of the **(1)** extra virgin olive oil (EVOO) and **(2)** extra virgin olive oil with rosemary (EVOO+R). **(B):** Effect of the different flavored EVOO (at a concentration of 7.5 mg/ml.) on gastric ulcer induced by EtOH/HClin Wistar rats.**(1)** Normal animals showing absence of ulcers; **(2)** ethanol-HCl alone administered control animals showing severe ulceration as reddish mucosal lesions; **(3)** standard drugs (ranitidine; 60 mg/kg bw. and Omeprazole; 30 mg/kg bw.) treated animals with less intense ulceration; **(4)** animals treated withRLC; **(5)** animals treated withOC; **(6)** animals treated withEVOO+R displaying minor ulceration; **(7)** animals treated withO+R showing mild ulceration with short mucosal lesions; **(8)** animals treated withEVOO+O; **(9)** animals treated withO+O; **(10)** animals treated withEVOO+L; **(11)** animals treated withO+L. **(C):** Principal component analysis (PCA) based on the chemical composition and biological activities (antioxidant, enzymatic, analgesic and gastroprotective) of the differentflavored olive oils. Object scores were highlighted for a bettervisualization.

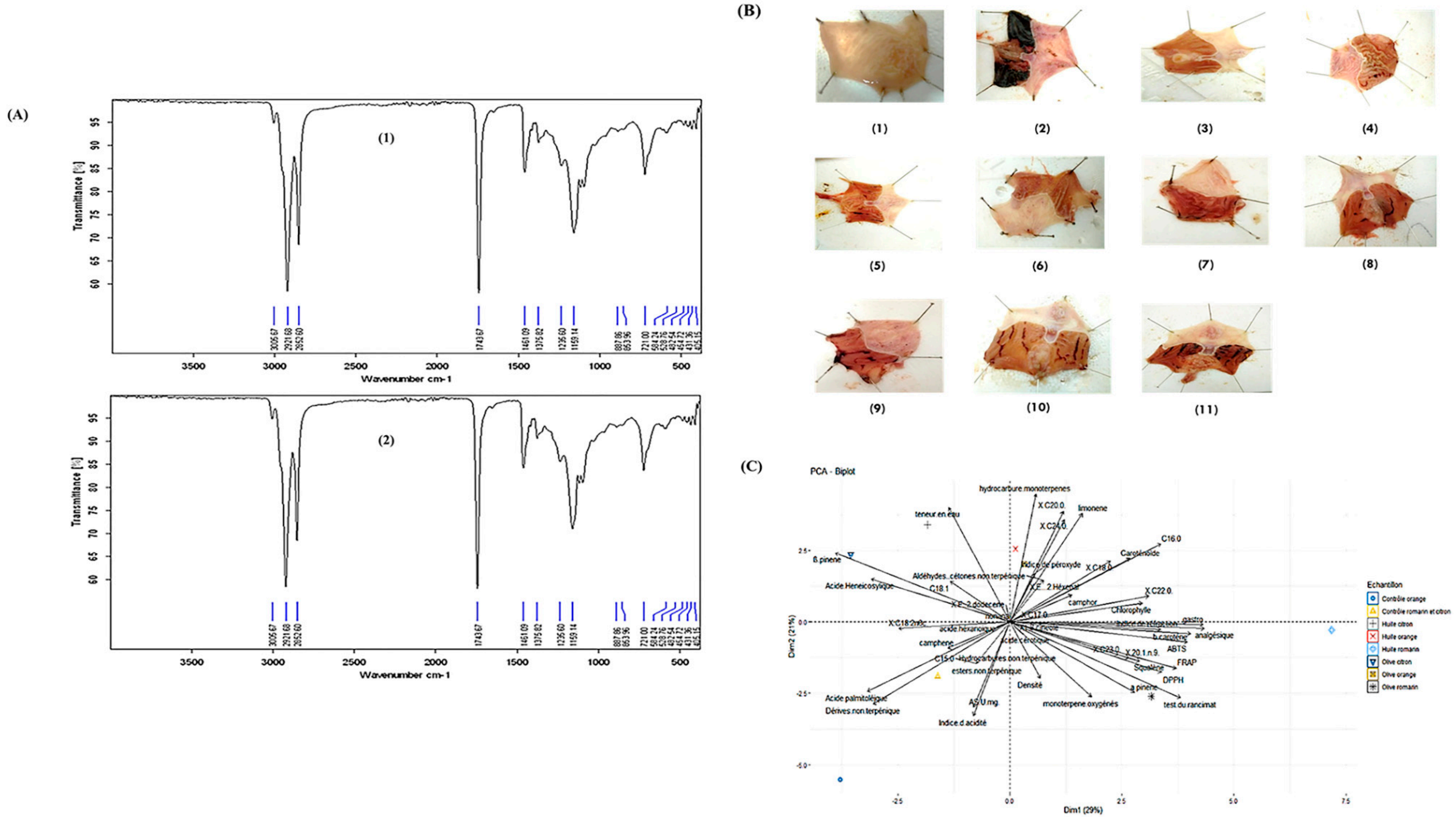
(A)
(1)
(2)
Transmittance [%]
Wavenumber cm-1
3005.67
2921.68
2852.60
1743.67
1461.09
1375.82
1235.60
1159.14
887.86
853.96
721.00
(B)
(1)
(2)
(3)
(4)
(5)
(6)
(7)
(8)
(9)
(10)
(11)
(C)
PCA - Biplot
Dim1 (29%)
Dim2 (21%)
Echantillon
Contrôle orange
Contrôle romarin et citron
Huile citron
Huile orange
Huile romarin
Olive citron
Olive orange
Olive romarin
hydrocarbure.monoterpenes
limonene
teneur.en.eau
C16.0
Caroténoïde
ß.pinene
Acide.Heneicosylique
C18.1
camphor
Chlorophylle
gastro
analgésique
ABTS
FRAP
DPPH
Squalène
test.du.rancimat
monoterpene.oxygénés
Densité
Indice.d.acidité
Acide.palmitoléique
Dérivés.non.terpénique
camphene
esters.non.terpénique
